# A Polymer Lithium-Oxygen Battery

**DOI:** 10.1038/srep12307

**Published:** 2015-08-04

**Authors:** Giuseppe Antonio Elia, Jusef Hassoun

**Affiliations:** 1 Department of Chemistry, Sapienza University, Piazzale Aldo Moro 5, 00185 Rome, Italy

## Abstract

Herein we report the characteristics of a lithium-oxygen battery using a solid polymer membrane as the electrolyte separator. The polymer electrolyte, fully characterized in terms of electrochemical properties, shows suitable conductivity at room temperature allowing the reversible cycling of the Li-O_2_ battery with a specific capacity as high as 25,000 mAh g_C_^−1^ reflected in a surface capacity of 12.5 mAh cm^−2^. The electrochemical formation and dissolution of the lithium peroxide during Li-O_2_ polymer cell operation is investigated by electrochemical techniques combined with X-ray diffraction study, demonstrating the process reversibility. The excellent cell performances in terms of delivered capacity, in addition to its solid configuration allowing the safe use of lithium metal as high capacity anode, demonstrate the suitability of the polymer lithium-oxygen as high-energy storage system.

Increasing global warming triggered urgent need for the reduction of greenhouse-gas emission and pollution, that may be effectively achieved by replacing conventional combustion-engine vehicles by sustainable electric or hybrid vehicles^1^,^2^. Lithium-air batteries represent one of the most appealing candidates for efficient electrified mobility due to its high energy density, in principle comparable to fossil fuels^3^,^4^,^5^. Lithium air battery operates through a complex mechanism, involving the reversible reduction of oxygen to superoxide anion O_2_^−∙^ in aprotic solvents^6^,^7^, and following lithium peroxide formation at the electrode/electrolyte interphase^8^. However, this system still suffers by several issues, such as the low stability of the conventional electrolytes against Li/O_2_ reaction products, the high cell polarization with consequent low energy efficiency and the short cycle life^9^,^10^,^11^. Indeed, efficient operation of the lithium oxygen battery requires a suitable electrolyte media^12^. Besides the systems efficiently employing new liquid electrolytes^13^,^14^,^15^,^16^, solid-state and polymer lithium oxygen battery attract a great attention^17^. These emerging systems may actually allow a safe use of the high-energy lithium metal, without flammability risks and associated safety issues due to possible dendrite formation, short-circuit and consequent thermal runaway^18^,^19^. Recently, several systems employing solid, polymer, gelled and hybrid electrolytes have been proposed^20^,^21^,^22^,^23^,^24^. In particular, lithium polymer battery is, in principle, a versatile system that may be laminated to form of thin film of plastic configuration by using the so-called *roll-to-roll* technique. Among them, polyethyleneoxide (PEO)–based electrolytes may be efficiently used in a lithium oxygen cell characterized by limited polarization, high reversibility and remarkable safety content due to the stability of the PEO-ether linkage against the reactive O_2_^−·^ radicals and the limited presence of side reactions^23^,^24^. However, the low room-temperature conductivity of this class of electrolytes, i.e. typically with value ranging from 10^−5^ to 10^−6^ S cm^−1^, in addition to the high interface resistance of the solid electrolyte hinder its applicability^25^,^26^. Plasticized polymer electrolyte may represent a valid alternative with improved characteristics, both in terms of conductivity and of interface resistance^27^.

In this work, we employ a plasticized, polyethyleneoxide (PEO)-based solid electrolyte, following indicated by the acronym PPE, for application in high energy lithium-oxygen battery. The electrolyte has a room-temperature conductivity of about 10^−3^ S cm^−1^ and shows excellent cell performance in terms of maximum delivered capacity, i.e., as high as 25 Ah g^−1^ (12.5 mAh cm^−2^). The reversibility of the electrochemical formation and dissolution of the lithium peroxide during cell operation is confirmed by ex-situ X-Ray diffraction. The cell using the PPE evidences a galvanostatic cycling with polarization as low as 0.6 V at 500 mAh g^−1^ capacity, with an energy efficiency close to the 80%. The results here reported suggest the applicability of the PEO-based solid electrolyte membrane in a high performances lithium-oxygen battery operating at room temperature. The solid configuration of the electrolyte and the absence of volatile and flammable components are expected to strongly limit possible safety issues and allow the use of the high capacity metallic lithium anode.

## Results and Discussion

**Figure 1:**
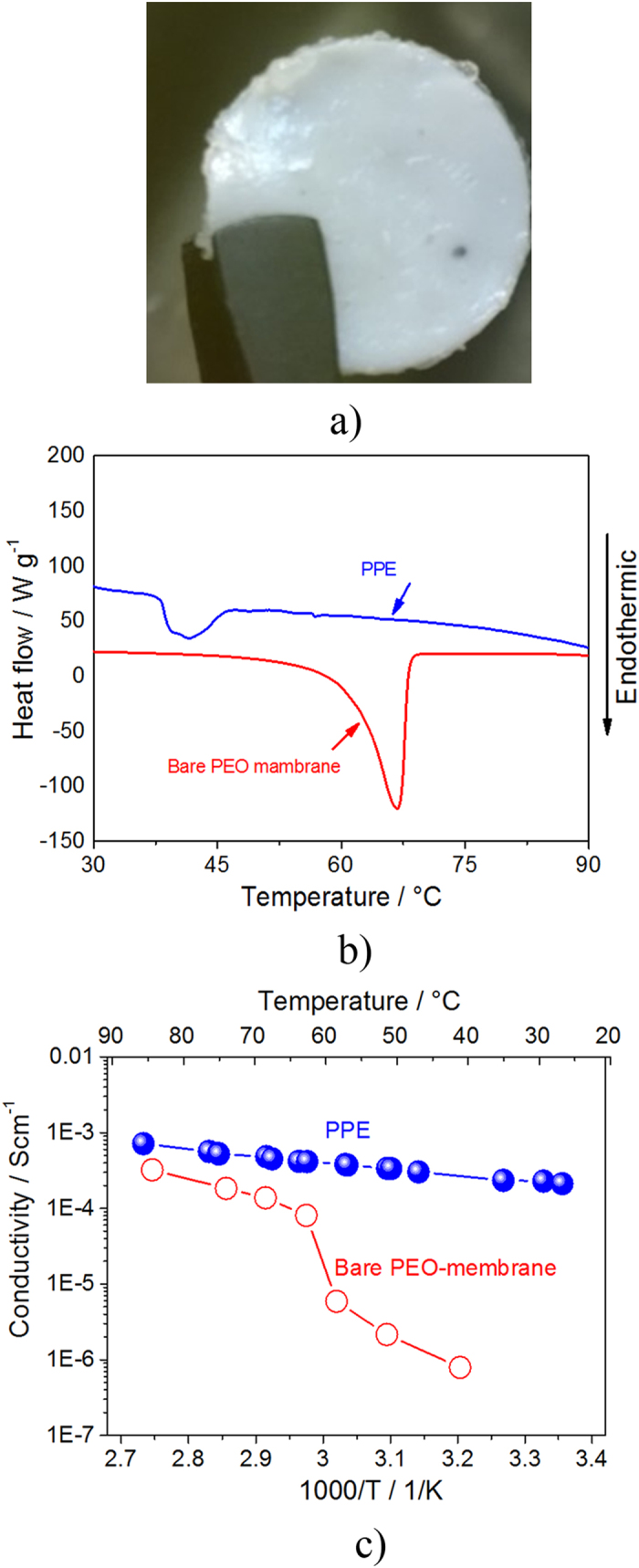
(**a**) Photographic image of the plasticized polymer electrolyte (PPE). (**b**) Differential Scanning Calorimetry (DSC) traces and (**c**) Arrhenius plots within 25 °C and 95 °C of the of the bare PEO_20_LiCF_3_SO_3_-ZrO_2_ membrane (red curves) and of the plasticized polymer electrolyte (PPE, blue curves).

The plasticized PPE membrane reported in the photographic image of Fig. 1a shows solid shape and good mechanical stability, allowing its direct application in coin-type lithium-oxygen cell^16^. Figure 1b, reporting the DSC traces of the pristine PEO-based membrane (red curve) and of the plasticized PPE (blue curve), respectively, evidences that the typical endothermic peak at about 65 °C, associated to the polymer melting and corresponding conductivity increase, shifts to about 40 °C by the plasticization process^28^. Furthermore, the integration of the peaks in Fig. 1b reveals a decrease of the melting enthalpy from 98.10 J g^−1^ for bare PEO membrane to 2.2 J g^−1^ for PPE electrolyte, thus suggesting a remarkable reduction of the membrane crystallinity by plasticization process. The melting temperature and enthalpy decrease is expected to increase the PPE conductivity, in particular at the lower temperature values, as indeed demonstrated by the Arrhenius plots reported in Fig. 1c. The conductivity trend of the bare-PEO membrane (Fig. 1c, red curve) evidences a typical behaviour characterized by values of the order of 10^−4^ S cm^−1^ at temperature higher than 60 °C and abrupt drop to values lower than 10^−5^ S cm^−1^ below 60 °C, due to the excessive membrane crystallization, already evidenced by the corresponding DSC traces. The PPE electrolyte (Fig. 1c, blue curve) shows different behaviour, with an almost linear trend and conductivity values ranging from 3·10^−4 ^S cm^−1^ to about 10^−3^ S cm^−1^, thus confirming the low crystallinity degree of the swelled membrane observed by DSC and suggesting the suitability of the adopted plasticization process for application in lithium battery^27^.

**Figure 2:**
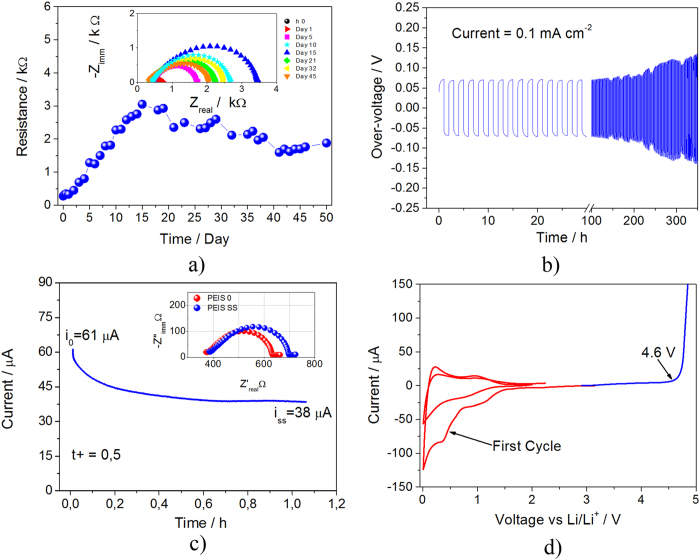
(**a**) Evolution of the resistance value in a symmetrical Li/PPE/Li cell upon storage time, in inset the corresponding Nyquist plots. (**b**) Voltage profile of the Li-stripping deposition measurement performed using a symmetrical Li/PPE/Li cell at a current of 0.1 mA cm^−2^ and a deposition stripping time of 1 hour. (**c**) Current vs. time profile of a symmetrical Li/PPE/Li upon 30 mV of polarization used for the determination of the Li-transference number, in inset the Nyquist plot of the cell before and after the polarization. (**d**) Electrochemical stability window of the PPE determined by cyclic voltammetry of a lithium/PPE/Super-C65(Cu) cell in the 0.01–2 V potential range (red curve) and linear sweep voltammetry of a lithium/PPE/Super-C65(Al) cell up to 4.8 V (blue curve). Scan rate 0.1 mV s^−1^, room temperature.

Figure 2 reports the characteristics of the PPE in terms of lithium interphase properties and electrochemical stability. The Li/PPE resistance evolution during time reported in Fig. 2a, and the corresponding impedance Nyquist plots in inset, evidence an initial resistance increase, extended to the first 15 days of measurement, followed by a decrease and a stabilization to values of about 2 kΩ. This trend is ascribed to the formation of a solid electrolyte interphase (SEI) film at the lithium surface due to partial reaction with the electrolyte and its subsequent consolidation and stabilization^29^. Figure 2b, reporting the lithium stripping-deposition galvanostatic test of a symmetrical Li/PPE/Li cell, shows relatively low polarization value, i.e. limited to about 0.1 V, that slightly increases to about 0.15 V after 200 hours of continuous cycling, as most likely associated to the increase of the interface resistance. Lithium transference number (t_Li+_) is a crucial parameter in determining an optimized lithium-cell behaviour. The t_Li+_ has been measured by following the Bruce-Vincent-Evans method consisting in the application to symmetrical Li/PPE/Li cell of a DC signal, the measurement of the current flowing through the cell and of the impedance values before and after the polarization, see Fig. 2c. According to this method, the lithium transference number may be calculated using the equation (1) 30:


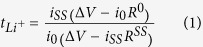


where ΔV is the applied signal amplitude, *R*^*0*^ and *R*^*ss*^ are the overall cell resistance values at the lithium electrode in the pristine state (before polarization) and at steady state condition (after polarization), *i*_*0*_ and *i*_*ss*_ are the current values measured immediately after polarization and at steady state condition. The application of the Bruce-Vincent equation indicated a t_Li+_ value of 0.5, reflecting a good ion mobility within the solid polymer electrolyte^25^.

Fig. 2d shows the electrochemical stability window of the PPE, as measured by voltammetric technique. The anodic scan reveals a stability against oxidation extended up to 4.6 V vs. Li/Li^+^, while the cathodic scan indicates an irreversible process during the first cycle, at potential ranging from 0.8 V to 0.2 V vs. Li/Li^+^ that is most likely ascribed to SEI formation at the carbon working-electrode surface preventing further electrolyte decomposition during the following cycles. At the lower potentials, i.e., within 0.2 V and 0.01 V vs. Li/Li^+^, the cyclic voltammetry shows the reversible Li-carbon electrochemical insertion process^31^.

**Figure 3:**
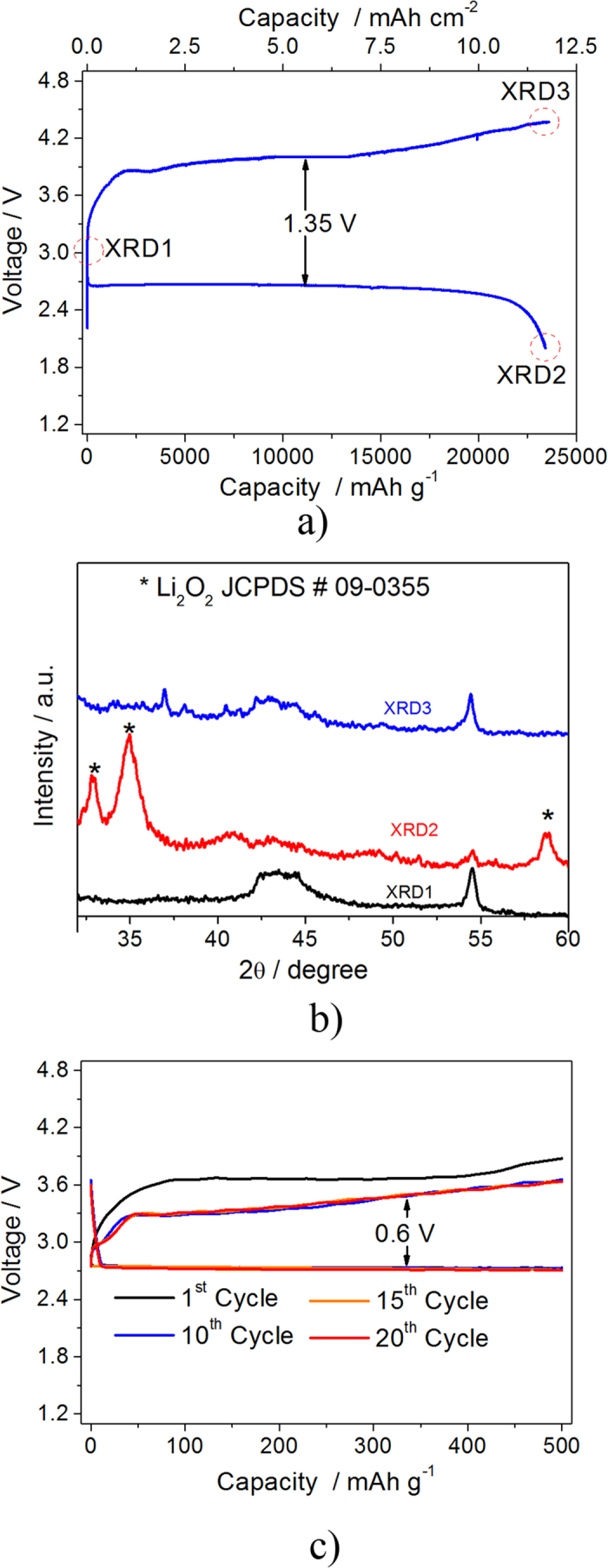
(**a**) Voltage profile of the galvanostatic cycling test of the lithium-oxygen polymer cell performed using a current of 200 mA g^−1^ within 2 V–4.3 V voltage range. (**b**) X-ray diffraction patterns of the gas diffusion layer used as the support for the Li_2_O_2_ deposition during lithium-oxygen cell operation collected at the pristine state of the cell (black pattern, XRD1), after full discharge (red pattern, XRD2) and full charge (blue pattern, XRD3). (**c**)Voltage profiles of the galvanostatic cycling test performed using a lithium oxygen polymer cell operating at controlled capacity regime by applying a 100 mA g^−1^ current with capacity limited to 500 mAh g^−1^.

The reversibility of the lithium-oxygen electrochemical process in cell employing the PPE has been studied by coupling XRD analysis and cycling test. Figure 3a shows the galvanostatic discharge-charge behaviour within 2 V and 4.3 V of the polymer lithium-oxygen cell. The figure shows a discharge capacity as high as 25,000 mAh g^−1^ referred to the carbon weight, i.e., 12.5 mAh cm^−2^ referred to the electrode surface area that is almost completely reversed during the following charge. The capacity value obtained by the Li/PPE/O_2_ cell is one of the highest referred to the carbon weight if compared to similar systems using conventional carbons^20^,^32^,^33^, as indeed evidenced by table T1 and figure S1, reporting a literature overview comprising a series of lithium-oxygen cells characterized by very high capacity in Supplementary Information section.

The relatively high capacity obtained adopting our cell configuration may be reasonably explained by considering a favorable lithium peroxide reaction mechanism within the PEO-based electrolyte media, mainly due to its high donor number^34^,^35^. Indeed, electrolytes characterized by high donor number allow proper growth of lithium peroxide both at the electrode surface as well within the electrolyte solution, while electrolytes having low donor number limit the lithium peroxide deposition reaction to the electrode surface^36^,^37^. Accordingly, an electrolyte favoring the Li_2_O_2_ growth from the electrode and within the solution, such as the system used in this work, leads to higher capacity in respect to a media in which the reaction occurs exclusively at the electrode surface, in particular considering the insulating nature of the formed products. Further measurements, aimed to determine the reproducibility of the result (see Figure S2 in Supplementary Information section), have indicated an error bar of about 8%. The relatively high cell polarization, i.e. of about 1.35 V, reflects the insulating nature of lithium peroxide covering in large amount the carbon electrode surface upon full discharge, as indeed evidenced by the ex-situ X-ray diffraction in Fig. 3b. The XRD diffraction of the discharged electrode (XRD2, red pattern) shows the peaks associated to the crystalline Li_2_O_2_ (marked by asterisks, JCPDS # 09–0355), while the recharged electrode (XRD3, blue pattern) reveals only few residual peaks, associated to the carbon electrode and to some electrolyte residual, evidencing the complete dissolution of the Li_2_O_2_ (compare XRD3 and XRD1 of the pristine electrode). These data show the formation and following dissolution of the lithium peroxide and demonstrate the reversibility of the electrochemical process. Furthermore, the remarkable reduction of the intensity of the peak at 54°, ascribed to the carbon support, upon full discharge indicates a relevant coverage of the electrode surface by the insulating Li_2_O_2_ and accounts for the notable cell polarization. The increase of the intensity of the peak ascribed to the carbon support upon the following full charge (XRD3) is in line with the Li_2_O_2_ electro-oxidation and consequent removal from electrode surface. Side reactions associated to the presence of an electrode electrolyte interphase cannot be completely excluded. Indeed, recent papers evidenced the presence in minor amount of decomposition products, not affecting the lithium oxygen cell behavior, in electrolytes formed by a mixture of low-molecular-weight PEO, esters, formates, oxymethylene and methyl methanoate^24^,^38^ However, the XRD patterns resolution as well as the presence of the impurities in a minor ratio hinder their proper detection in our cell configuration.

The high cell polarization issue may be effectively mitigated by limiting the amount of the Li_2_O_2_ deposited during the discharge process, thus allowing the carbon component to improve the electrode conductivity. This enhancement may be achieved by cycling the lithium oxygen polymer cell under limited capacity regime, as demonstrated by Fig. 3c reporting the galvanostatic test of a cell cycled limiting the capacity to 500 mAh g^−1^ (Current 100 mA g^−1^). The figure shows a first cycle characterized a polarization value of about 0.9 V followed by stable cycles with a low polarization limited to 0.6 V, with an energy efficiency close to the 80%. The change of the voltage profile may be reasonably attributed to several factors, including the SEI film formation at the electrode surface and modifications of the carbon structure and morphology within the electrode by repeated cycling^39^. Indeed, recent paper evidenced the possible morphological change of the carbon employed as cathode support due to its direct reaction with the lithium-oxygen reaction products^40^. Following the first cycle, the voltage profile stabilizes and the cell exhibits excellent cycling behaviour and enhanced energy efficiency, thus further confirming the suitability of the PPE for application in advanced, safe lithium-oxygen polymer battery.

## Conclusions

A polymer configuration lithium-oxygen battery is here reported. The battery evidences a high reversibility and an improved energy efficiency in respect to the typical Li/O_2_ systems in view of an optimized electrode/ electrolyte interface characteristics. In addition, the battery has a high safety level due to its solid polymer configuration. The cell shows a maximum capacity of 25,000 mAh g^−1^ referred to the carbon electrode weight reflecting in a surface capacity of 12.5 mAh cm^−2^. Considering a discharge voltage of 2.7 V and a reversible capacity limited to 500 mAh g^−1^, the theoretical energy density of the cell may be calculated as 1350 Wh kg^−1^ that is considered a high value, in particular if compared to the conventional lithium-ion battery cathodes, typically ranging from 600 to 700 Wh kg^−1^. It is expected that this high theoretical energy value may reflect in a practical energy higher than 300 Wh kg^−1^ upon cell optimization, i.e. a value two times higher than that associated to the conventional lithium ion battery.

## Methods

### Polymer membrane preparation

The plasticized polymer electrolyte (PPE) was obtained by swelling a PEO_20_LiCF_3_SO_3_ + 10 % ZrO_2_ membrane in a tetraethylene glycol dimethyl ether (TEG-DME) 1 m LiCF_3_SO_3_ solution for 24 hours at 50 °C. The PEO_20_LiCF_3_SO_3_ + 10 % ZrO_2_ polymer matrix (bare PEO-electrolyte) was prepared using a procedure described in a previous paper^25^,^26^,^27^. Briefly, the polyethyleneoxide (PEO 600000 Mw Aldrich) and lithium trifluoromethanesulfonate (Aldrich LiCF_3_SO_3_), in a Oxygen/Lithium molar ratio of 20:1, and 10% w:w of ZrO_2_ ceramic powder (Aldrich,) were mixed for 24 hours, using the low energy glass milling system. The mixture was following hot pressed in a 90 cm^−2^ surface area aluminum plate at 90 °C, at 0.5 tons for 15 min and 4.0 ton for 45 min. The TEG-DME 1m LiCF_3_SO_3_ solution, used for membrane plasticization, was obtained by dissolving lithium trifluormethanesulfonate (LiCF_3_SO_3_ 99.9% Sigma Aldrich) in tetraethylene glycol dimethyl ether (TEGDME 99% Sigma Aldrich). PEO, LiCF_3_SO_3_ and ZrO_2_ ceramic filler were dried under vacuum for 2 day at 55 °C, at 110 °C and 130 °C, respectively. The TEG-DME was purified using dry zeolite 4 Å, until the water content was below 10 ppm. All the preparation procedures were performed in an argon filled glove box, with a water and oxygen content lower than 1 ppm.

### Electrolyte characterization

The characteristics of the PPE were investigated in terms of thermal behavior, ionic conductivity, stability against lithium, ionic transference number and electrochemical stability window. Differential Scanning Calorimetry (DSC) was performed within 25 °C and 95 °C using Mettler Toledo DSC instrument. The ionic conductivity of the PPE was evaluated by impedance spectroscopy in a frequency ranging from 200 kHz to 10 Hz, applying a 10 mV amplitude signal in a 2032-coin cell using a Teflon spacer of known size in order to fix the cell constant, in a 25–95 °C temperature range. The lithium transference number of the membrane was evaluated using the Bruce Vincent method^30^, applying a 30 mV DC-signal and determining the initial and the steady state current, and the corresponding impedances by the support of electrochemical impedance spectroscopy (EIS). The lithium/electrolyte interphase proprieties were investigated in terms of stability against lithium in stationary condition. The test consisted in the evaluation of the impedance of a symmetrical lithium/lithium cell during storage time. The impedance measurements were performed within frequency ranging from 200 kHz to 10 mHz by applying a 10 mV BIAS. The stability of the lithium/electrolyte interphase in dynamic condition was evaluated by a stripping/deposition measurement using a symmetrical Li/PPE/Li cell employing a current of 0.1 mA cm^−2^ and a deposition-stripping time of 1 hour. The anodic stability of the electrolyte was evaluated by linear sweep voltammetry with a scan rate of 0.1 mV s^−1^, using a Super-C65 working electrode coated on aluminum. The cathodic stability was determined by cyclic voltammetry in a 0.01–2 V potential range at 0.1 mV s^−1^ scan rate employing a Super-C65 working electrode coated on copper foil.

### Lithium-oxygen polymer cell preparation and testing

The lithium-oxygen polymer cell was cycled using a 2032 meshed coin cell^16^ with lithium metal as the anode, the PPE as the separator and a gas diffusion layer coated by carbon as the support for oxygen reaction. This electrode has been obtained using a gas diffusion layer GDL 35 BC as support (SGL Group) with a thickness of 315 μm, a porosity of the 80%, an air permeability of 1 cm^3^/(cm^2^ s) and an electrical resistance (through plane) ≤ 12 mΩ cm^2^. A slurry comprising 80% Super C65 (Imerys, surface area of about 62 m^2^ g^−1^) and 20% PvdF (6020 Solef Solvay), in N-methyl-pyrrolidinone (NMP Sigma Aldrich), has been cast onto the GDL obtaining a final carbon loading of about 0.5 mg cm^−2^. The dry slurry thickness was of about 15 microns. The galvanostatic cycling of the lithium oxygen cell was performed within 2V and 4.3 V, using a current of 200 mA g^−1^. The reaction mechanism of the full cell was investigated by ex-situ X ray diffraction of the gas diffusion layer collected from pristine, full discharged and full charged cell. Prior measurement, the electrode was washed several times with the dimethyl carbonate (DMC, Merck battery grade, water content less than 10 ppm) in order to remove electrolyte traces. The XRD measurement was performed by Rigaku D-Ultima Max diffractometer using a Cu kα radiation source. Capacity controlled galvanostatic cycling tests were performed by applying a current of 100 mA g^−1^ limiting the capacity to 500 mAh g^−1^. All the electrochemical measurements were performed using a VSP Biologic instrument.

## Additional Information

**How to cite this article**: Elia, G. A. and Hassoun, J. A Polymer Lithium-Oxygen Battery. *Sci. Rep.* **5**, 12307; doi: 10.1038/srep12307 (2015).

## Supplementary Material

Supplementary Information
