# Supplementary material for: A Polymer Lithium-Oxygen Battery
Source: Sci Rep. 2015 Aug 4;5:12307. doi: 10.1038/srep12307 (PMC4523859; doi:10.1038/srep12307)
Supplement: Supplementary Information [file srep12307-s1.pdf]

A Polymer Lithium-Oxygen Battery

Giuseppe Antonio Elia and Jusef Hassoun*

Department of Chemistry, Sapienza University, Piazzale Aldo Moro 5, 00185 Rome, Italy
Corresponding author: jusef.hassoun@uniroma1.it

SUPLLEMENTARY INFORMATION

Table T1 and corresponding figure S1 report a literature overview comprising a series of lithium-oxygen cells characterized by very high capacity (Light red: cells using conventional carbon based cathode; Light blue: cell using cathodes comprising catalyst; Grey: cells using various carbon morphology i.e. CNTs, RGO, ...etc). Figure S1 shows that the configuration here adopted allows the highest gravimetric capacity obtained using conventional, carbon based electrodes, while the surface capacity appears of the same order of magnitude. However, this value does not represent the highest value reported in literature for lithium oxygen battery. Indeed, the figure shows that the use of a carbon nanotube based cathode allows the achievement of a capacity as high as 56800 mAh g^{-1} corresponding to a surface capacity of 45.44 mAh cm^{-2}. Furthermore, the figure evidences that a slightly lower gravimetric capacity in respect to the one obtained by our cell configuration, i.e. of the order of 15000 mAh g^{-1} and higher surface capacity, i.e. of about 30 mAh cm^{-2}, may be obtained by increasing the carbon loading. The capacity obtained by a lithium oxygen battery is in principle determined by the lithium amount used at the anode side, in excess of oxygen. Indeed, we used a 14 mm-diameter lithium disk (18 mg of Li metal) corresponding to a capacity of 47 mAh cm^{-2} assuming the full formation of Li_2O_2. This capacity, when referred to the carbon weight used in our cathode side corresponds to 72000 mAh g^{-1}_c. However, the insulating nature of the formed products at the cathode hinders the achievement of such as high capacity. In addition, low cell polarization as well as increased cycle life and stability of the lithium oxygen battery may be achieved only by limiting the ratio of lithium peroxide deposited at the cathode as indeed demonstrated by our paper (see fig 3c) and by literature results (refs. 16, 33, 34)..

Reference	Specific Capacity referred to the carbon [mAh g^{-1}]	Surface capacity [mA cm^{-2}]
Wang, F et al. A dual pore carbon aerogel based air cathode for a highly rechargeable lithium-air battery. J. Power Sources 272, 1061 (2014).	13500	9.54
Luo, W. B., Chou, S. L., Wang, J. Z., Zhai, Y. C., & Liu, H. K. A facile approach to synthesize stable CNTs@ MnO electrocatalyst for high energy lithium oxygen batteries. Scientific reports 5 (2015).	6500	7.15
Wang, X., A Li–O_2/Air Battery Using an Inorganic Solid-State Air Cathode. ACS Appl. Mater. Interfaces 6, 11204 (2014).	14192	9.50
Zhang, T., & Zhou, H. A reversible long-life lithium–air battery in ambient air. Nature communications 4, 1817 (2013). (Cycling test in open Air)	56800	45.44
Zhang, T., & Zhou, H. A reversible long-life lithium–air battery in ambient air. Nature communications 4, 1817 (2013). (Cycling test in Argon atmosphere plus trace of Air)	19050	15.24
Jung, Hun-Gi, et al. A transmission electron microscopy study of the electrochemical process of lithium–oxygen cells. Nano letters 12, 4333 (2012).	10000	10
Han, S. M., Kim, J. H., & Kim, D. W. Cycling Performances of Lithium-Air Cells Assembled with Mixed Electrolytes of Ionic Liquid and Diethylene Glycol Diethyl Ether J. Electrochem. Soc. 162, A3103 (2015).	11000	11
Xiao, J, et al. Hierarchically porous graphene as a lithium–air battery electrode. Nano letters 11, 5071 (2011).	15000	30
Luo, W-B, et al. A Metal-Free, Free-Standing, Macroporous Graphene@ g-C3N4 Composite Air Electrode for High-Energy Lithium Oxygen Batteries. Small (2015) doi: 10.1002/smll.201403535.	17500	35
Liu, Y, et al. Novel approach for a high-energy-density Li–air battery: tri-dimensional growth of Li_2O_2 crystals tailored by electrolyte Li^+ ion concentrations. Journal of Materials Chemistry A 2 9020 (2014).	13245	9.27
Sun, B., Munroe, P., Wang, G. Ruthenium nanocrystals as cathode catalysts for lithium-oxygen batteries with a superior performance. Scientific reports 3 (2013).	10000	10
Black, R., Lee, J. H., Adams, B., Mims, C. A., & Nazar, L. F. The role of catalysts and peroxide oxidation in lithium–oxygen batteries. Angewandte Chemie, 125, 410 (2013).	14000	1.75
Our cell	23034	11.51
Li, Y., Wang, J., Li, X., Geng, D., Banis, M. N., Li, R., & Sun, X. Nitrogen-doped graphene nanosheets as cathode materials with excellent electrocatalytic activity for high capacity lithium-oxygen batteries.Electrochemistry Communications 18, 12 (2012)..	11500	4.84
Xu, J. J., Xu, D., Wang, Z. L., Wang, H. G., Zhang, L. L., Zhang, X. B. (2013). Synthesis of Perovskite-Based Porous $La_{0.75}Sr_{0.25}MnO_3$ Nanotubes as a Highly Efficient Electrocatalyst for Rechargeable Lithium–Oxygen Batteries. Angewandte Chemie International Edition, 52(14), 3887-3890.	11000	9.9

Table T1 Gravimetric capacity referred to the carbon weight and corresponding surface capacity of various lithium oxygen cells reported in literature, using different configurations (Light red: cells using conventional carbon based cathode; Light blue: cell using cathodes comprising catalyst; Grey: cells using various carbon morphology i.e. CNTs, RGO, ...etc)

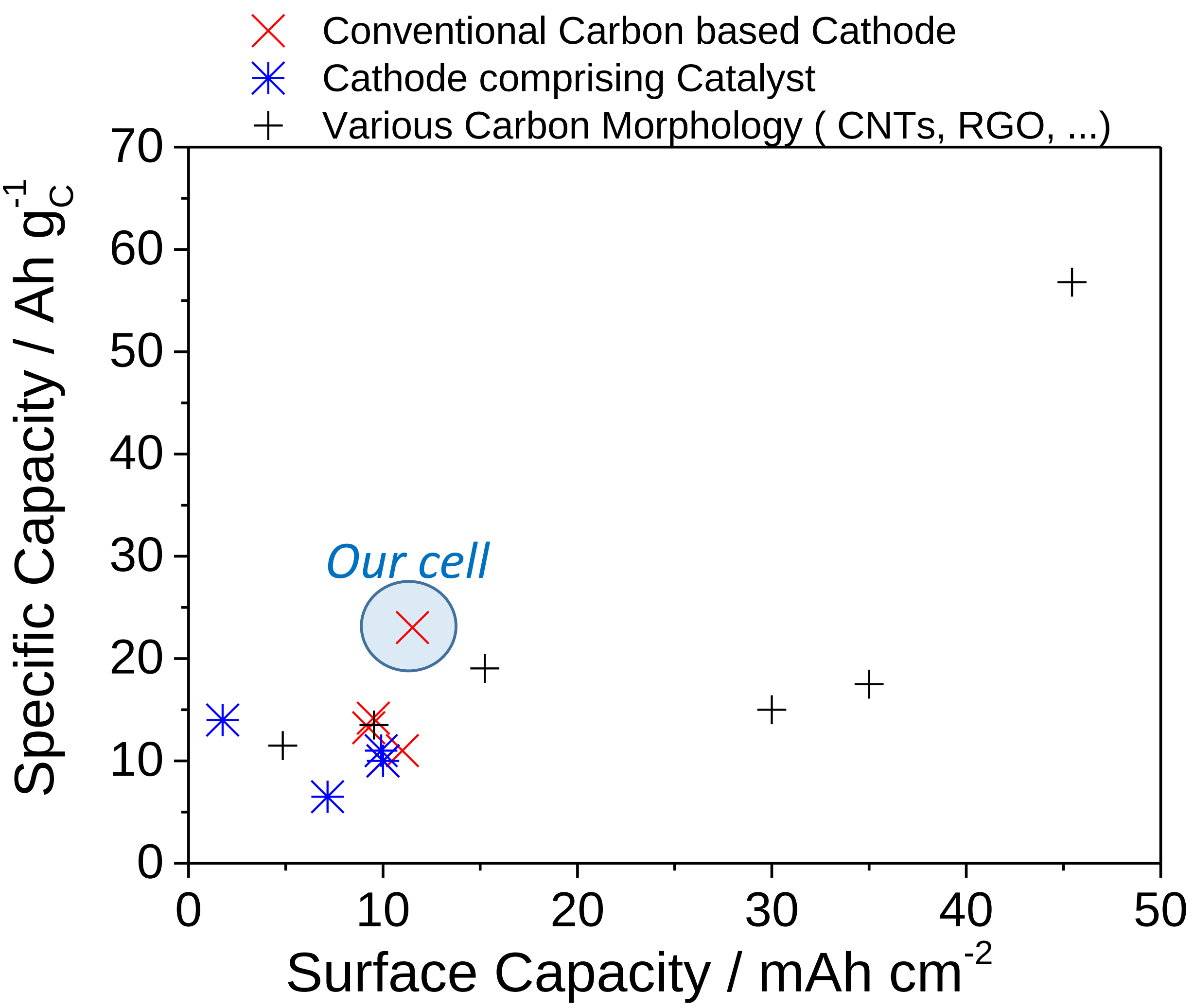

Figure S1 Specific gravimetric capacity vs. surface capacity of various lithium oxygen cells reported in literature and using different configurations (see table T1 for corresponding references).

The reproducibility of our battery has been determined by repeated measurements, performed using tree electrodes with the same thickness in lithium-oxygen polymer cells. The results reported in Figure S2 show a capacity ranging from about 20000 mAh g^{-1} to 23000 mAh g^{-1}, thus suggesting an average capacity of about 21500 mAh g^{-1} within a standard deviation of 1750 mAh g^{-1} , i.e. about 8%.

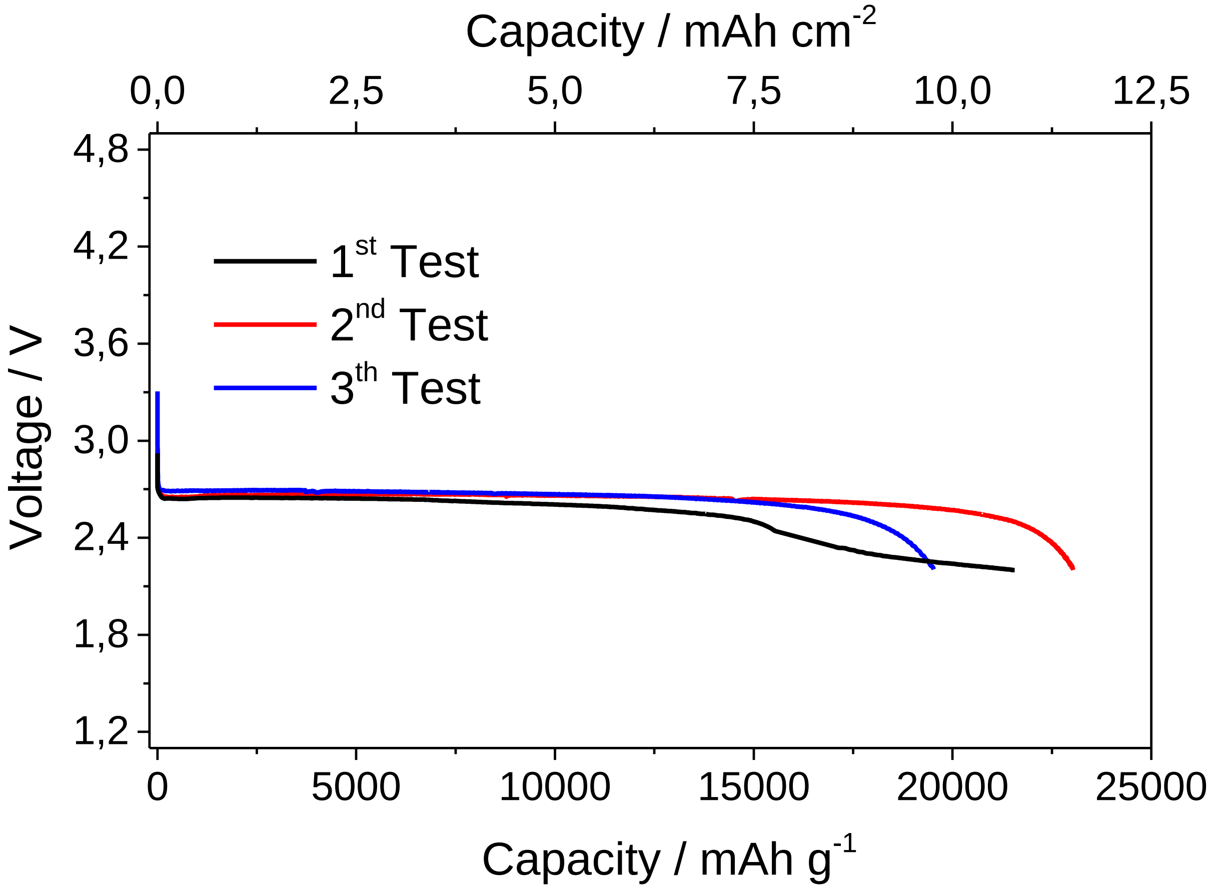

Figure S2 Voltage profile of the galvanostatic discharge of the lithium-oxygen polymer cell performed using a current of 200 mA g^{-1} down to 2V. The measurement has been repeated in order to determine the reproducibility of the test.
